# Oxytocin Versus Oral Misoprostol for Induction of Labor in Pregnant Women with Term Prelabor Rupture of Membranes: a Randomized Clinical Trial

**DOI:** 10.1007/s43032-023-01290-0

**Published:** 2023-07-13

**Authors:** Rania Hassan Mostafa Ahmed, Mohamed Samir Eid Sweed, Gasser Adly El-Bishry, Raghda Khaled Hassan

**Affiliations:** Department of Gynecology and Obstetrics, Faculty of Medicine, Ain Shams University, Cairo, Egypt

**Keywords:** Oxytocin, Misoprostol, Cesarean section, Vaginal delivery, Chorioamnionitis

## Abstract

**Abstract:**

This study compares the effectiveness and safety of oxytocin infusion against oral misoprostol for inducing labour in pregnant women with term prelabor membrane rupture. We randomized 173 pregnant women presenting with term prelabor rupture of membranes (PROM) at Ain Shams University Maternity Hospital into Group A (underwent induction of labor (IOL) by 25μg misoprostol oral tablet every 4 h, for maximum 5 doses) and an identical Group B: (underwent IOL by oxytocin infusion according to the hospital protocol). Our primary outcome was rate of vaginal delivery within 24 h, while the secondary outcomes included the time till active phase, induction to delivery interval, maternal pyrexia, nausea and vomiting, fetal distress, Apgar score, birth weight, and neonatal intensive care unit admission. Both groups showed high rates of vaginal delivery (82.4% & 87.1% for misoprostol group and oxytocin group respectively) with no significant difference between the two groups (*p*=0.394). However, patients induced by misoprostol took significantly less time to reach active phase with a shorter induction to delivery interval as compared to patients induced with oxytocin. This difference was clear in multiparous women, but not observed in primiparous women when subgroup analysis was done. No significant difference was found as regards other outcomes. Our study showed that both oral misoprostol and oxytocin are effective and safe for IOL in patients with PROM, with shorter induction-delivery interval in patients induced by oral misoprostol, an effect that is clear in multiparous but not primiparous women.

**Trial registration:**

NCT05215873, on 31/01/2022, “retrospectively registered”.

**Supplementary Information:**

The online version contains supplementary material available at 10.1007/s43032-023-01290-0.

## Introduction

It is common practice to induce labour in an effort to enhance maternal and infant health outcomes and lower incidence of caesarean births, prolonged labour, gestational hypertension, and postpartum haemorrhage [1]. In 2017, rates of labour induction reached up to 25% in the USA and 33% in Australia, with differences between nations [1].

Prelabor rupture of the membranes (PROM) occurs when the fetal membranes rupture before the onset of labor. About 8% of term pregnancies are complicated by this, and the most serious maternal consequence is intrauterine infection, the risk of which rises with the duration of the membrane rupture [2].

A 2017 Cochrane systematic review comparing expectant care for PROM at term vs planned early delivery (with induction of labour) found that planned early birth reduced the risk of maternal infectious morbidity without appearing to increase the chance of caesarean section [3].

In comparison to other widely used techniques (oxytocin, Foley catheter, amniotomy), the use of prostaglandin for cervical ripening in women with low Bishop scores has been shown to be more effective [4]. Nevertheless, uterine tachysystole, hyperstimulation syndrome, alterations in foetal heart rate (FHR), and uterine rupture are all more common [5]. Despite that, prostaglandins, including misoprostol, are still recommended for induction of labour (IOL), and result in fewer cesarean sections than oxytocin alone, as concluded by the Cochrane systematic review [6]. This conclusion was reached by Pourali et al. in their study, which found that sublingual misoprostol for IOL in PROM cases is more efficient than oxytocin and has superior neonatal outcomes [7]. Misoprostol taken orally or sublingually is an appealing option for IOL since it is affordable, heat stable, and simple to use [6, 7].

On the other hand, oxytocin, the most widely used induction agent globally, is the agent of preference suggested in cases of PROM by the American College of Obstetricians and Gynecologists [2], likely due to its higher safety profile. Moreover, the study by Kulhan found that oxytocin was more efficient than vaginal dinoprostone in achieving vaginal birth within 24 h of induction in term pregnancies with PROM [8].

Thus, it is still a clinical challenge to identify the most secure, efficient, and simple to deliver induction drug in PROM cases. This study aims to compare the efficacy and safety of oral misoprostol versus oxytocin infusion for IOL in pregnant women with PROM at term.

## Methods

This randomized controlled clinical trial was conducted in Ain Shams University Maternity Hospital (ASUMH), Cairo, Egypt over the period of one year (January 2021 to January 2022). The study protocol was approved by the ethical committee, faculty of Medicine, Ain Shams University (FMASU MS 733/2020/2021). The study was conducted in accordance with the Declaration of Helsinki and retrospectively registered at ClinicalTrials.gov (NCT05215873). Informed consent was obtained from all participants before recruitment in the study. All data was collected confidentially.

We included pregnant women between the ages of 18 and 40 who had term (gestational age: 36 to 42 weeks) PROM during the previous 24 h, had vertex presentation, and were scheduled for IOL at ASUMH. Multiple pregnancies, antepartum haemorrhage, chorioamnionitis/PROM lasting longer than 24 h, abnormal FHR pattern upon admission, intrauterine growth restriction, foetal malpresentation, estimated foetal weight greater than 4 kg, patients already in labour, women with a history of uterine scarring, current or past medical conditions, and known allergies to prostaglandin/oxytocin were among the exclusion criteria. We included the gestational age (36 weeks) in our study as per our hospital’s protocol, we follow active management and delivery for patients 36 weeks and beyond with PROM. As per hospital protocol, patients received parenteral antibiotic Unasyn® 1.5g every 8h till delivery.

Eligible patients were randomized using a computer-generated sequence 1:1 either to the Misoprostol group (group A) or to Oxytocin group (group B). Randomization was done using computer generated randomization sheet using MedCalc® version 13. Accordingly, patients were allocated to either: Group A: received 25μg misoprostol oral tablet every 4 h, for maximum 5doses to induce labour; while—according to the ASUMH oxytocin protocol—women in group B underwent IOL by oxytocin infusion as follows: Oxytocin Mix: 3IU oxytocin (3000mIU) + 50ml of normal saline in syringe pump (60mIU/ml), to start at 1ml/hour (1mIU/min) for half-hour, then shift to 2ml/h for half-hour if contractions weren't strong enough with reassuring FHR. When FHR was reassuring but still insufficient contractions, we raised by 2 ml/hour every half-hour until a maximum of 27 ml/h. Follow-up throughout labour included continuous electronic FHR monitoring, a vaginal examination every 4 h to check for cervical changes, maternal vital signs, and uterine contractions. After noting the time until the active phase (cervical dilatation: 6 cm), follow-up monitoring continued till delivery.

End point: reaching maximum dose of drugs with failure of IOL in which another decision was taken. Also, at any point there was foetal or maternal distress (e.g., pathological FHR pattern, antepartum hemorrhage, etc.) the study intervention was stopped, and the maternal/fetal condition was managed accordingly.

Our primary outcome was the percentage of successful vaginal deliveries within 24 h. The secondary outcomes were the time from the beginning of induction to the active phase (6 cm cervical dilatation), the induction to delivery interval, the maternal side effects of pyrexia, nausea, and vomiting, foetal distress (hyperstimulation), the Apgar score, the birth weight, and the admission to the neonatal intensive care unit (NICU).

### Sample Size Justification

Sample size was calculated using PASS 11® program, setting alpha error at 5%, and power at 80%. Earlier findings from the trial by Zeterglu et al. [9] demonstrated that the expected mean interval from induction to delivery in misoprostol group=10.61 ± 2.45 h as compared to 11.57 ± 1.91 h in oxytocin group. Hence, the required sample size was calculated to be 85 women per group.

### Statistical Analysis

The collected data was revised, coded, tabulated, and introduced to a PC using Statistical Package for Social Science (SPSS® 25). Numerical data was described by mean and standard deviation, or median and interquartile range. Categorical data was expressed by frequency and percentage. Student *t* test and Mann-Whitney test were used to assess the statistical significance of the difference between two study group means. Chi square test and Fishers’ exact test were used to examine the relationship between categorical variables. A *P* value < 0.05 was considered statistically significant.

## Results

**Fig. 1:**
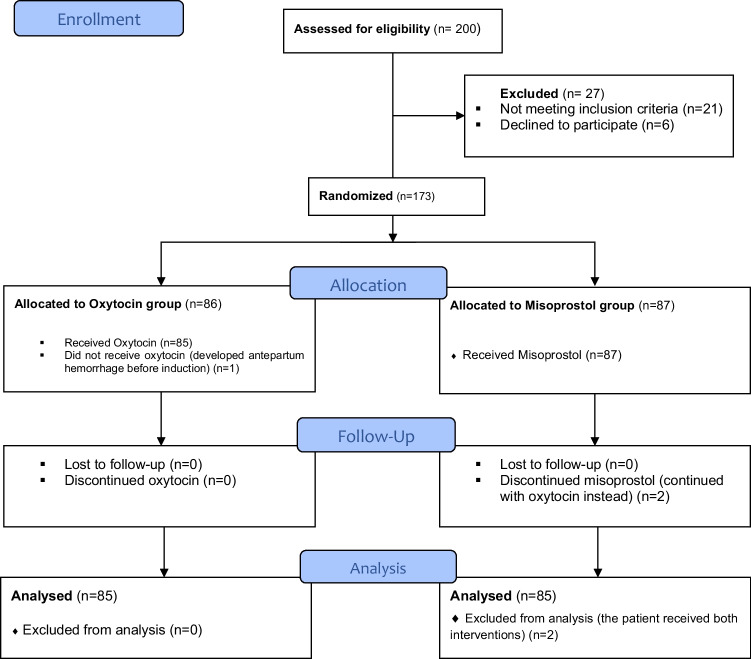
CONSORT diagram showing the recruitment and follow-up of patients during the study

Two hundred pregnant women with term PROM attending at ASUMH for IOL were recruited from the labor ward and assessed for eligibility; of which, a total of 173 women were enrolled and randomized and allocated into two groups: Misoprostol group (*N*=87) and Oxytocin group (*N*=86). The process of recruitment and follow-up of the patients during the study is shown in the CONSORT diagram (Fig. 1).

### Section I: Demographical and Clinical Characteristics of Both Groups

Demographical and baseline clinical characteristics of patients in both study groups are shown in Table 1. Patients in both groups were similar as regards age, body mass index, parity, mean arterial pressure, as well as gestational age and bishop score upon admission.

**Table 1:** Demographical and clinical characteristics of the study groups

		Misoprostol group		Oxytocin group		*t* test		
		Mean	SD	Mean	SD	*T*	*p* value	sig.
Age (years)		25.59	4.13	25.02	3.74	*t* = 0.935	0.351	NS
Body mass index (kg/m2)		26.83	3.05	27.35	3.52	*t* = -1.030	0.305	NS
Gestational age (weeks)		37.94	1.47	38.11	1.40	*t* = -0.747	0.456	NS
Mean arterial pressure (mmHg)		92.24	8.33	92.41	8.11	*t* = -0.140	0.889	NS
		Median	IQR	Median	IQR	Mann-Whitney test		
Bishop score on admission		4	4-4	4	3–4	*Z*= 1.276	0.202	NS
		N	%	N	%	Fisher exact test		
Parity	0	18	21.2%	16	18.8%	X^2^ =3.1	0.212	NS
	1	36	42.4%	47	55.3%			
	2	31	36.5%	22	25.9%			

### Section II: Analysis of Different Outcomes in Both Study Groups

As regards mode of delivery, both groups showed high rates of vaginal delivery (82.4% & 87.1% for misoprostol group and oxytocin group respectively) with no significant difference between the two groups (*p*=0.394). However, there was a significant difference in time taken to deliver in the two groups. Patients induced by misoprostol took significantly-less time to reach the active phase (5.86 vs 6.74; *p*<0.001), and a significantly lower induction to delivery interval (7.5 vs 8.43; *p*<0.001), as compared to patients induced by oxytocin, as shown in Table 2.

**Table 2:** Mode and time of delivery in both groups

		Misoprostol group		Oxytocin group		Test of sig.		
		*N*	%	*N*	%	value	*p* value	sig.
Mode of delivery	Vaginal	70	82.4%	74	87.1%	X^2^ =0.73	0.394	NS
	CS	15	17.6%	11	12.9%			
reason for Cesarean	Abnormal CTG pattern	9	60.0%	6	54.5%	Fisher exact test	0.746	NS
	Failed induction	4	26.7%	2	18.2%			
	Arrest of labor	2	13.3%	3	27.3%			
		Mean	SD	Mean	SD	t test		
Time till active phase (hours)		5.86	1.08	6.74	1.33	*t*=-4.331	<0.001	**S**
Induction to delivery interval (hours)		7.50	1.27	8.43	1.53	*t*=-3.967	<0.001	**S**

Both groups had similar neonatal outcomes, as there was no significant difference between babies born in both groups as regards birth weight, Apgar scores at 1 and 5 min, and percentage of babies admitted to NICU. Also, both oxytocin and misoprostol seem to be safe agents for IOL, as hyperstimulation only occurred in 3 cases with misoprostol and 2 cases with oxytocin, with no significant difference between them, as shown in Table 3. Maternal side effects were also minimal and comparable in both groups as shown in Table 4.

**Table 3:** Neonatal outcomes of both groups

		Misoprostol group		Oxytocin group		*t* test		
		Mean	SD	Mean	SD	*t*	*p* value	sig.
Birth weight (grams)		3306.47	218.19	3277.65	231.91	0.835	0.405	NS
Apgar score at 1 minute		6.06	1.37	6.46	1.51	-1.812	0.072	NS
Apgar score at 5 minutes		8.06	1.18	8.19	1.21	-0.706	0.481	NS
		N	%	N	%	Fisher exact test		
NICU admission	Absent	82	96.5%	81	95.3%		1.000	NS
	Present	3	3.5%	4	4.7%			
Hyperstimulation	Absent	82	96.5%	83	97.6%		1.000	NS
	Present	3	3.5%	2	2.4%

**Table 4:** Maternal side effects in both groups

		Misoprostol group		Oxytocin group		Fisher exact test	
		*N*	%	*N*	%	*p* value	sig.
Pyrexia	Absent	79	92.9%	84	98.8%	0.117	NS
	Present	6	7.1%	1	1.2%		
Nausea	Absent	81	95.3%	81	95.3%	1.000	NS
	Present	4	4.7%	4	4.7%		
Vomiting	Absent	84	98.8%	83	97.6%	1.000	NS
	Present	1	1.2%	2	2.4%		
PPH	Absent	82	96.5%	84	98.8%	0.621	NS
	Present	3	3.5%	1	1.2%		

### Section III: Subgroup Analysis

We performed subgroup analysis; trying to look whether the same results apply if we sub-grouped the cases according to the parity (whether primiparous or multiparous). As regards the mode of delivery, the results were the same: no significant difference between oxytocin and misoprostol in both subgroups (primiparous and multiparous groups). However, as regards the time taken to reach active phase and induction to delivery interval; the subgroups were different. Among primiparous women, there was no significant difference between misoprostol and oxytocin, in contrast to multiparous women; where misoprostol group took significantly-less time to reach active phase and had a shorter induction to delivery interval. This is shown in Tables 5 and 6.

**Table 5:** Mode of delivery among primiparous and multiparous women in both groups

Among primiparous women		Misoprostol group		Oxytocin group		Fisher exact test		
		*N*	%	*N*	%	*p* value		sig.
Mode of delivery	Vaginal	17	94.4%	16	100.0%	1		NS
	CS	1	5.6%	0	0.0%			
Among multiparous women		Misoprostol group		Oxytocin group		Chi square		
		*N*	%	*N*	%	X^2^	*p* value	sig.
Mode of delivery	Vaginal	53	79.1%	58	84.1%	0.556	0.456	NS
	CS	14	20.9%	11	15.9%

**Table 6:** Time to deliver among primiparous and multiparous women in both groups

	Misoprostol group		Oxytocin group		t test		
	Mean	SD	Mean	SD	*T*	*p* value	sig.
Among Primiparous women							
Time till active phase (hours)	5.75	1.10	6.23	1.42	-1.085	0.286	NS
Induction to delivery interval (hours)	7.67	1.53	7.74	1.52	-0.138	0.891	NS
Among Multiparous women							
Time till active phase (hours)	5.90	1.08	6.88	1.28	-4.345	0.001	S
Induction to delivery interval (hours)	7.44	1.19	8.62	1.49	-4.576	0.001	S

## Discussion

This randomized trial aimed to compare the efficacy and safety of oral misoprostol versus oxytocin infusion for IOL in pregnant women with term PROM. The study revealed that both misoprostol and oxytocin are effective and safe for IOL. However, we found that patients induced by misoprostol took significantly-less time to reach active phase with a shorter induction to delivery interval as compared to patients induced with oxytocin. This difference was clear in multiparous women, but not observed in primiparous women when subgroup analysis was done.

IOL has been managed using a variety of techniques, both medicinal and mechanical, each having advantages and disadvantages. Prelabor ROMs is a unique indication for IOL, and we need a technique that works quickly to prevent prolonged ROMs and potential foetal distress or intraamniotic infection.

Misoprostol, a synthetic prostaglandin E1 analogue, is employed to promote uterine contractions and cervical ripening. It has the benefits of being affordable, readily available, and heat stable [10, 11]. It can be administered orally, sublingually, vaginally, or rectally [11]. Since we were targeting patients with PROM in our study, we opted the oral route to prevent the potential of infection. The Cochrane systematic review concluded that using oral misoprostol for IOL is as effective as vaginal misoprostol and vaginal dinoprostone in achieving vaginal birth, and results in fewer cesarean sections if compared to oxytocin alone [6].

Throughout labour and the postpartum period, oxytocin naturally promotes uterine contractions. Its synthetic analogues are commonly used for both induction and augmentation of labour [10]. The American College of Obstetricians and Gynecologists recommends it as the preferred agent for IOL in PROM cases, but at least 12–18 h of adequate uterine contractions should be allowed for the latent phase of labor to progress before diagnosing failed induction and moving to cesarean delivery [2].

In our study, there was no significant difference between the two agents in obtaining high rates of vaginal delivery (82.4% and 87.1% for the misoprostol group and the oxytocin group, respectively). This was similarly observed in the review by Lin et al as they concluded that misoprostol is an effective and safe agent for IOL in women with term PROM, and when compared with oxytocin, the risk of hyperstimulation and the rate of maternal and neonatal complications were similar [12]. However, the review by Mozur et al found that prostaglandin E2 and vaginal misoprostol were more effective than oxytocin in bringing about vaginal delivery within 24 h but were associated with more uterine hyperstimulation [13]. Moreover, a 2014 Cochrane review found that oral misoprostol for IOL is safe, efficient, and results in fewer caesarean deliveries than oxytocin alone [6]. Nevertheless, we must keep in mind that these two reviews did not specify cases of PROMs, instead they included studies for IOL in general. On the other hand, Kulhan et al. compared oxytocin to dinoprostone (prostaglandin E2 analogue) for IOL in nulliparous women with term PROMs and found that oxytocin is more effective in achieving vaginal delivery within 24 h (64.3% vs 47.3% for oxytocin and dinoprostone respectively) [8].

As regards the time taken to deliver, we found that misoprostol group had shorter total induction to delivery interval, an effect that was noted only in multiparous and not primiparous women when subgroup analysis was employed. When Unthanan et al. compared sublingual misoprostol to oxytocin for IOL in 170 pregnant women with term PROM, they came to similar conclusions. They found that induction time and cesarean section rates of sublingual misoprostol group were significantly lower than the intravenous oxytocin group [14]. On the other hand, Freret et al studied a retrospective cohort of 130 term, nulliparous women with PROM who underwent IOL with intravenous oxytocin or buccal misoprostol, and they concluded that intravenous oxytocin was associated with faster admission-to-delivery times than buccal misoprostol [15]. These contradictory findings concerning the time required for induction and delivery may be the result of different dosages being employed, particularly for the protocol of oxytocin administration and titration. Also, there’re some differences noted when dealing with nulliparous or multiparous women, which is not unified in all studies.

Although IOL in cases with PROM is a bit different from IOL in cases without PROM (as the local prostaglandins released might affect the degree of the cervical ripening and might have a role in augmenting the uterine contractions); it is worth mentioning some results of other studies comparing misoprostol to oxytocin in IOL in cases without PROM. De Aquino and Cecatti compared misoprostol to oxytocin for IOL in term pregnant women with intact membranes. They randomized 210 patients to either intravaginal 25μg misoprostol every 4 h (maximum 8 doses) or intravenous oxytocin continuous infusion. They concluded that misoprostol was more efficient for cervical ripening and labor induction than oxytocin [16]. However, this was contradicting the results by Angela Wilson-Liverman, who conducted a randomized clinical trial comparing vaginal misoprostol 25 μg every 4 h up to a maximum of 4 doses, to oxytocin intravenous infusion, for IOL in multiparous women. They found that induction to delivery interval was shorter with oxytocin than misoprostol, however the difference was not statistically significant (*p* value 0.11) [17].

Our study’s key strength is that it exclusively included patients with PROM; thus, it would help to cover the gap of knowledge as regards the best method of IOL in this subset of patients with special situation being more susceptible to infection, and to possible hyperstimulation due to release of endogenous prostaglandins along with rupture of the membranes.

Our study does, however, have certain drawbacks. Although there was a statistically significant difference in the induction-delivery interval, its clinical significance is yet to be determined. We did not measure markers of possible infection in the neonates; only an overall assessment of the neonate by the Apgar score and NICU admission.

Another issue is that blinding was not applicable (both the physician and the patient knew which intervention was being used). However, this might not be a concern, as the outcomes we measured were not subjective, but objective outcomes not affected by bias, neither from the observer or the patient.

One other thing: in the group who were induced by oxytocin, the oxytocin infusion was maintained as such till delivery. As for the group induced by misoprostol, oxytocin was not used for augmentation of labour, and if used, the patient was excluded from the study analysis. We acknowledge that this might have implications on the time from active phase till delivery; however, the time from starting induction till reaching the active phase was also measured as one of the secondary outcomes, so it can be used to correctly compare the two interventions as head-to-head comparison. Starting IOL with misoprostol and continuing it with oxytocin is a widespread practice. Hence, more investigations are required to determine whether or not this will be superior. Another point which might have implications on the measures of time to delivery is the type and dose of the analgesic used during labour. Some patients received opiates, others did not. Unfortunately, this data was not collected (which patients took which drug in which dose). Whether this would change the results significantly or not, is not clear.

## Conclusions

In conclusion, our study showed that both oral misoprostol and oxytocin are effective and safe for IOL in patients with PROM, with shorter induction-delivery interval in patients induced by oral misoprostol, an effect that’s clear in multiparous but not primiparous women. We recommend oral misoprostol for IOL in multiparous women with PROM, to shorten the time of induction and possibly reduce the likelihood of intraamniotic infection.

### Supplementary Information

Below is the link to the electronic supplementary material.

Supplementary file1 (XLSX 31.9 KB)

## Data Availability

The dataset used and analyzed during the current study is available as supplementary file.

## Abbreviations

ASUMH: Ain Shams University Maternity Hospital
FHR: foetal heart rate
IOL: induction of labour
PROM: prelabor rupture of the membranes
NICU: neonatal intensive care unit
